# Relapse or Re-Infection, the Situation of Recurrent Tuberculosis in Eastern China

**DOI:** 10.3389/fcimb.2021.638990

**Published:** 2021-03-17

**Authors:** Yan Shao, Honghuan Song, Guoli Li, Yan Li, Yishu Li, Limei Zhu, Wei Lu, Cheng Chen

**Affiliations:** ^1^Department of Chronic Communicable Disease, Center for Disease Control and Prevention of Jiangsu Province, Nanjing, China; ^2^Department of Epidemiology and Statistics, School of Public Health, Southeast University, Nanjing, China

**Keywords:** recurrence, relapse, re-infection, MIRU-VNTR, tuberculosis

## Abstract

**Purpose:**

Recurrent tuberculosis (TB) is defined by more than one TB episode per patient and is caused by re-infection with a new *Mycobacterium tuberculosis* (Mtb) strain or relapse with the previous strain. Recurrence of TB is one important obstacle for End TB strategy in the world and elucidating the triggers of recurrence is important for the current TB control strategy in China. This study aimed to analyze the sources of recurrent TB by the molecular genotyping method.

**Method:**

A population-based surveillance was undertaking on all culture-positive TB cases in Jiangsu province, China from 2013 to 2019. Phenotypic drug susceptibility test (DST) by proportion method and mycobacterial interspersed repetitive units-variable number of tandem repeat (MIRU-VNTR) were adopted for drug resistance and genotype detection.

**Results:**

A total of 1451 culture-positive TB patients were collected and 30 (2.06%, 30/1451) TB cases had recurrent TB episodes. Except 7 isolates were failed during subculture, 23 paired isolates were assessed. After genotyping by MIRU-VNTR, 12 (52.17%, 12/23) paired recurrence TB were demonstrated as relapse and 11 (47.83%,11/23) paired cases were identified as re-infection. The average interval time for recurrence was 24.04 (95%CI: 19.37-28.71) months, and there was no significant difference between relapse and re-infection. For the relapsed cases, two paired isolates exhibited drug resistance shifting, while four paired isolates revealed inconsistent drug resistance among the re-infection group including two multidrug-resistant tuberculosis (MDR-TB) at the second episode.

**Conclusion:**

Relapse and re-infection contributed equally to the current situation of recurrence TB in Jiangsu, China. Besides, more efficient treatment assessment, specific and vigorous interventions are urgently needed for MDR-TB patients, considering obvious performance among re-infection cases.

## Introduction

Tuberculosis (TB) is an old communicable disease and the leading cause of death from a single infectious agent. Globally, it was estimated 10.0 million people fell ill with TB in 2018, and only 7.0 million TB patients were notified and reported to WHO as new or relapse cases. China still ranks in the 30 high TB burden countries with a total incidence rate of 61 per 100000 population in 2018 (WHO, 2019). Although China obtained tremendous successes in controlling the TB epidemic in the past years, it is not on track to reach the target of End TB Strategy yet. There are many challenges faced by clinical and national TB control programs to achieve the targets even with improvements in TB diagnosis, treatment and prevention. One such obstacle is the recurrence of TB which varied by country and region according to previous studies (Hozbor et al., 2017; Wingfield et al., 2019). A combined analysis of the mathematical model revealed that further reducing new TB cases only has a modest effect on disease burden, but interventions that restrain reactivation have a greater improvement on disease burden in China (Houben et al., 2016).

Either endogenous reactivation or exogenous infection could result in a new episode of TB even after a complete anti-TB treatment. Comparing the isolates from the first and second episodes of TB could distinguish such two different causes. It was commonly considered that two episodes with identical isolates as a relapse, otherwise, it means exogenous infection or namely re-infection. However, under special circumstances with a dominant cluster of TB strain, the possibility of a re-infection by identical genotypes could not be overlooked as well (Folkvardsen et al., 2020). In this study, patients with identical strain genotype were considered a potential relapse, otherwise a re-infection. In the high TB incidence region, re-infection would be the principal reason for recurrent TB (Bryant et al., 2013a). Genotyping methods for *Mycobacterium tuberculosis complex* (MTBC), such as spoligotyping and mycobacterial interspersed a repetitive units-variable number of tandem repeat (MIRU-VNTR) typing, which utilize variations in repetitive sequences in MTBC strains, enable researchers to discriminate relapse and re-infection (Oelemann et al., 2007). The different type of recurrent TB requires a specific control strategy. If re-infection accounted for the majority of TB recurrence, a powerful control strategy of infection control should be adopted. Otherwise, effective treatment of TB should be reinforced. Some studies have demonstrated recurrent TB in China by molecular epidemiology, but they proposed quite different results about the proportion of re-infection (Nsofor et al., 2017; Zong et al., 2018). So we conduct this retrospective study to evaluate relapse and re-infection among those recurrent TB patients in eastern China. Meanwhile, we adopted the profiles of demographic characteristics of TB cases and drug-resistant patterns of isolates to explore the potential effect on the recurrent TB.

## Methods

### Study Population

This study was undertaken in TB drug resistance surveillance spots in Jiangsu Province which were established according to national survey of drug-resistant TB (Zhao et al., 2012). During 2013-2019, all newly registered pulmonary TB patients with either sputum smear-positive or molecular testing positive were consecutively collected after an informed consent was obtained. Furthermore, sputum samples were performed culture on Lowenstein-Jensen (LJ) media as well. Finally, a total of 1451 culture-positive TB patients were collected, including new cases and previously treated cases (WHO, 2009). Those TB patients were followed up by the National Health Management and Information System (HMIS), and we found that 30 patients presented the second episode of TB. The isolates from recurrent TB patients which conserved with cryoprotectant at -80°C were thawed and re-cultured on L-J media in our provincial TB laboratory. Except 7 isolates were failed in the subculture, twenty-three pairs of cases were finally enrolled for further analysis. Meanwhile, the interval time between the first and second episodes and the demographic features were collected.

### Treatment and Follow-Up

Without obtaining the drug-resistant information, the treatment regimen was prescribed based on the history of TB treatment, and all the cases were classified into new cases and previously treated cases. All recruited new cases were treated with standard 2HRZE/4HR regimens, which started with 2 months of daily isoniazid (H), rifampicin (R), pyrazinamide (Z), and ethambutol (E), then followed by daily isoniazid (H) and rifampicin (R) for another 4 months. Meanwhile, for previously treated cases, streptomycin (S) was added to the four drugs (HRZE) daily for 2 months and then three drugs (HRE) daily lasting to 6 months (2HRZES/6HRE) (WHO, 2010). The detailed treatment regimens and durations were list in **Table 1**. We followed up all cases from treatment initiate to recurrence of TB during the study period.

**Table 1:** Drug resistance status and clinical information depending on recurrence type.

Recurrence type	NO. pair	Interval time (month)	Treatment regimen for the first episode	Drug Resistance Status		Chest X-ray of the first episode: cavitation
				First episode	Second episode	
re-infection	2	10	2HRZE/4HR	susceptible	susceptible	Yes
	3	33	2HRZES/6HRE	resistant to R,H,S	susceptible	Yes
	5	23	2HRZES/6HRE	susceptible	susceptible	No
	8	21	2HRZE/4HR	susceptible	susceptible	Yes
	11	22	2HRZES/6HRE	susceptible	susceptible	Yes
	14	45	2HRZE/4HR	susceptible	resistant to R,H,S	Yes
	15	16	2HRZES/6HRE	susceptible	resistant to R,H,S	No
	16	19	2HRZE/4HR	susceptible	susceptible	No
	18	38	2HRZE/4HR	resistant to H	resistant to H	No
	20	25	2HRZE/4HR	susceptible	resistant to H,S	Yes
	22	11	2HRZE/4HR	susceptible	susceptible	No
relapse	1	9	2HRZES/6HRE	susceptible	susceptible	No
	4	22	2HRZES/6HRE	susceptible	susceptible	Yes
	6	54	2HRZE/4HR	resistant to R	susceptible	No
	7	26	2HRZE/4HR	susceptible	susceptible	Yes
	9	26	2HRZE/4HR	susceptible	susceptible	Yes
	10	16	2HRZE/4HR	susceptible	susceptible	No
	12	26	2HRZE/4HR	susceptible	susceptible	No
	13	26	2HRZE/4HR	susceptible	susceptible	No
	17	24	2HRZE/4HR	susceptible	susceptible	Yes
	19	13	2HRZES/6HRE	resistant to H	resistant to R,H,E	No
	21	29	2HRZES/6HRE	susceptible	susceptible	Yes
	23	19	2HRZE/4HR	susceptible	susceptible	No

R, rifampicin; H, isoniazid; S, streptomycin; Z, pyrazinamide; E, ethambutol.

### Drug Susceptibility Test

All subcultured positive isolates were analyzed by phenotypic drug susceptibility test (DST) by proportion method on Lowenstein–Jensen (L-J) media (Baso, Zhuhai, China). The p-nitrobenzoic acid (PNB) test was adopted for *non-tuberculosis* mycobacterium (NTM) identification. DST performed on L-J media adopted the following critical drug concentrations: rifampicin 40µg/ml, isoniazid 0.2µg/mL, streptomycin 4µg/mL, and ethambutol 2µg/mL respectively (WHO, 2009).

### DNA Extraction

About two loops of the bacterial growth were scraped from L-J media (Baso, Zhuhai, China) and placed into the 500µl of 75% ethanol bath in a microcentrifuge tube. Then, the sample was sterilized in the 75% ethanol bath for 1 hour after 5 minutes of ultrasound. After centrifuging with 12,000 g for 3 min, the supernatant was discarded. The sample was resuspended in 200µl of 20mg/ml lysozyme solution (Sangon Biotech, Shanghai, China) with glass powder for DNA releasing thoroughly. The following steps were referred to chemical lysis with cetyltrimethylammonium bromide (CTAB) method. In general, CTAB-NaCl solution was added and incubated at 65°C for 10 minutes, then the mixture of Chloroform: isoamyl alcohol (24:1) mixture was added followed by a centrifuge of 12000 g for 5 min. Wash the sample again with Chloroform: isoamyl alcohol mixture and remove the upper phase to a new tube with cold isopropanol, after gentle mixture and the solution was frozen for at least 30 min. After thawing and washed with 70% cold ethanol, the solution was centrifuged for 20 minutes at 12000g. Finally, the supernatant was discarded and dried pellets were resuspended in 50µl of Tris-EDTA (TE) buffer (Yates et al., 2002).

### MIRU-VNTR Analysis

The standard 24 loci were performed for genotyping of *Mycobacterium tuberculosis* according to Supply et al. (2006) After amplification of extracted DNA, PCR products were examined by 1.5% agarose gel. To analyze the genetic relationship between the first and second episodes, the web application MIRU-VNTRplus was adopted, and the re-infection case was defined as paired isolates with different MIRU-VNTR patterns at two or more than two loci according to phylogenetic lineage identification (Interrante et al., 2015; Maghradze et al., 2019).

### Statistical Analysis

The questionnaire was double entered in EpiData 3.1 (EpiData Association, Odense, Denmark). Person chi-square test or Fisher’s exact test were used to compare the categorical variables, and *t*-test was applied for the continuous variables. All analyses were performed using SAS 9.3 software (SAS Institute, Inc., Cary, NC, USA) and *P*<0.05 was considered statistically significant.

## Results

**Figure 1:**
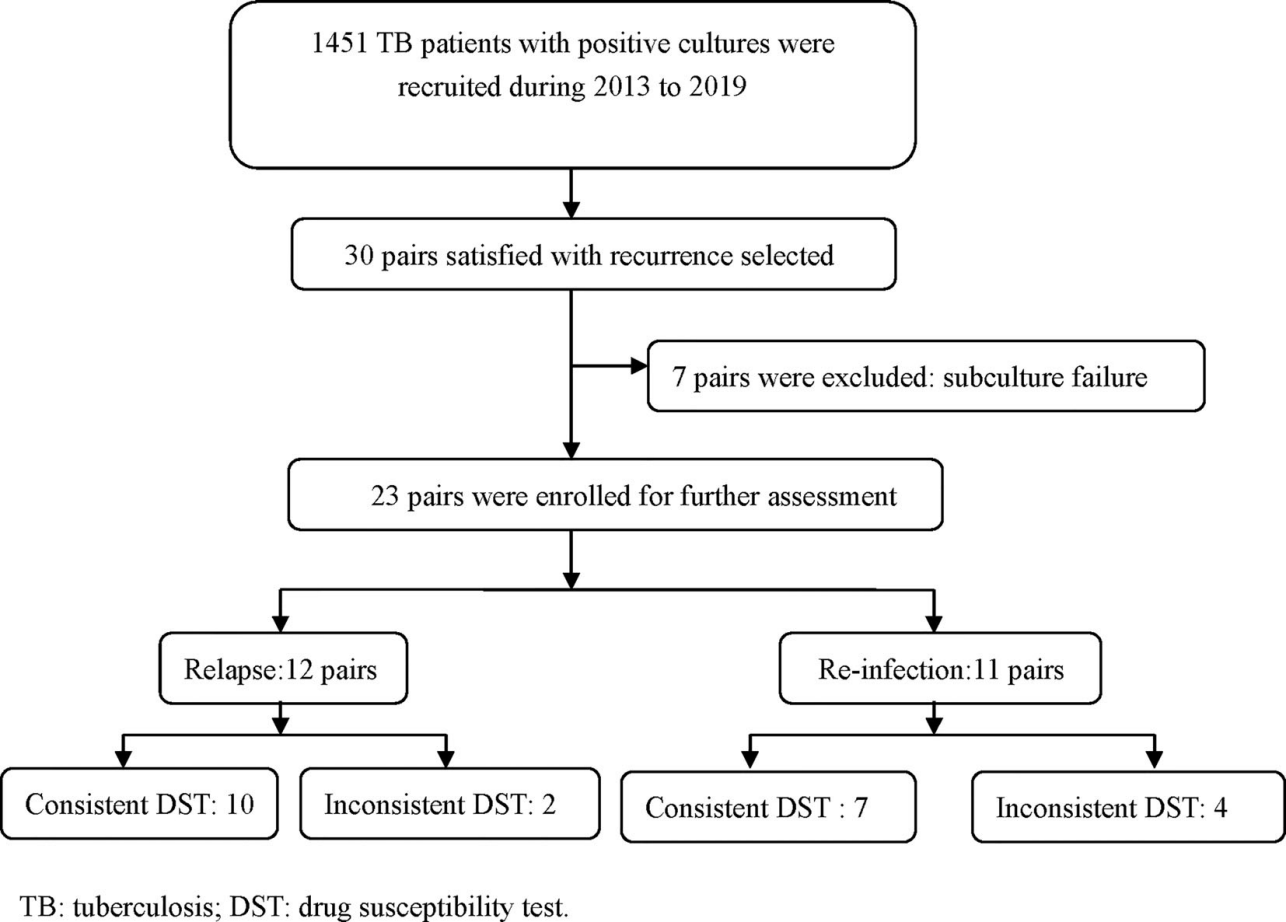
Study flow for case enrollment.

During the study period, a total of 1451 TB patients from tertiary hospitals provided positive cultures and up to 2% (30/1451) were observed recurrence after completion of anti-TB treatment. Except 7 isolates failed in the subculture, 23 pairs of isolates were undergoing the MIRU-VNTR method to distinguish the genotypes (**Figure 1**). For the 23 recurrent patients, the mean age was 49.48 ± 22.71 years, and the male gender accounted 82.60% (19/23). According to the treatment history, new cases accounted 65.22% (15/23) of the total. The chest X-ray indicated that 47.83% (11/23) recurrent TB patients had cavitation for the first episodes.

**Figure 2:**
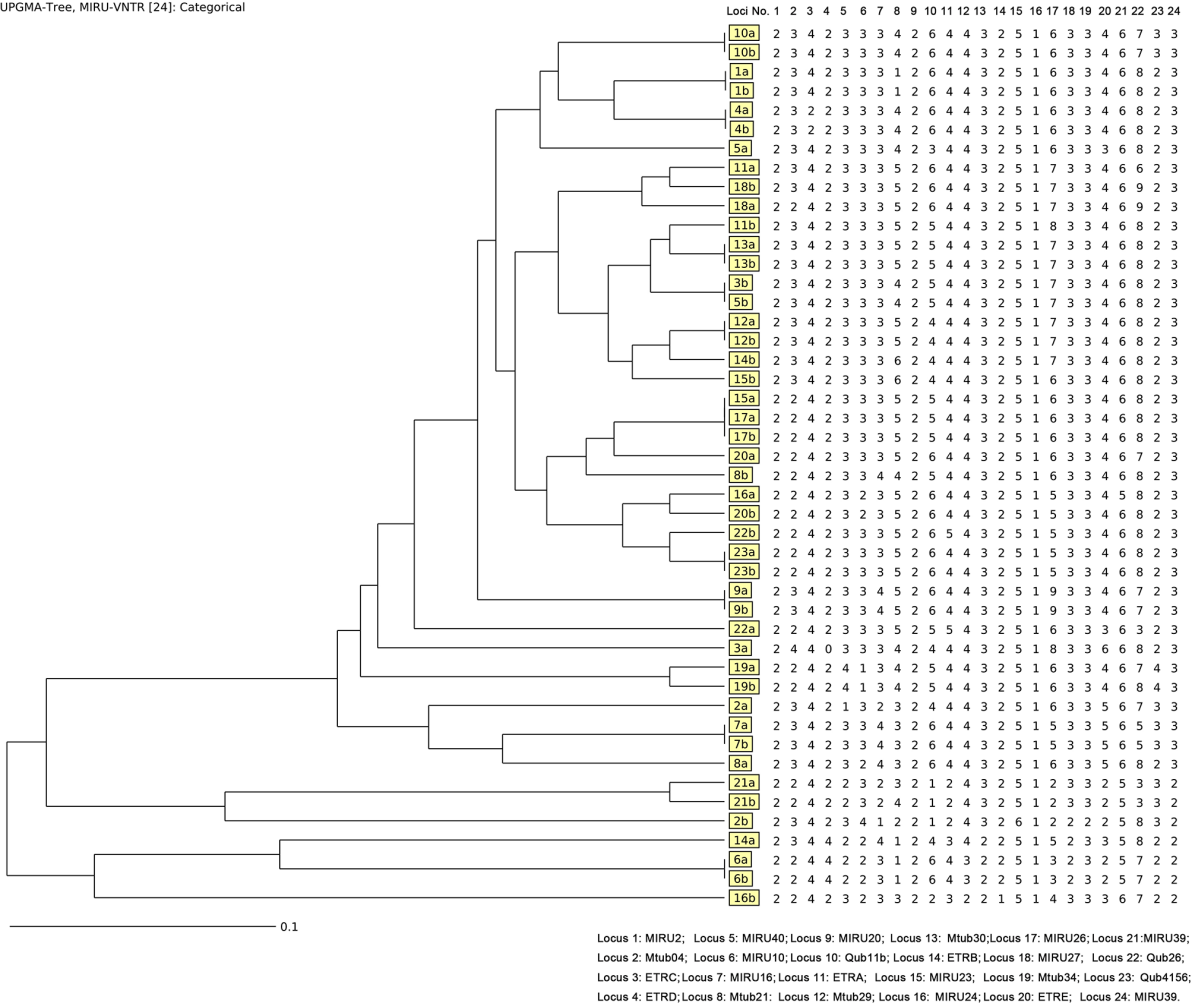
Genotype results for the 23 paired isolates by MIRU-VNTR.

Considering the power of discrimination, 24 loci were adopted in this study, and the genotype results based on MIRU-VNTR of each isolate were demonstrated in **Figure 2**. Out of 23 pairs of isolates, 12 (52.17%, 12/23) pairs exhibited consistent genotype patterns by the minimum spanning tree algorithm, while another 11 (47.83%,11/23) pairs of isolates exhibited different MIRU-VNTR patterns at more than two loci (**Figure 2**). There is no significant difference between relapse and re-infection for age (*t*=0.835, *P*=0.413), gender (*P*=0.640), and cavitation (*P*=0.381).

**Figure 3:**
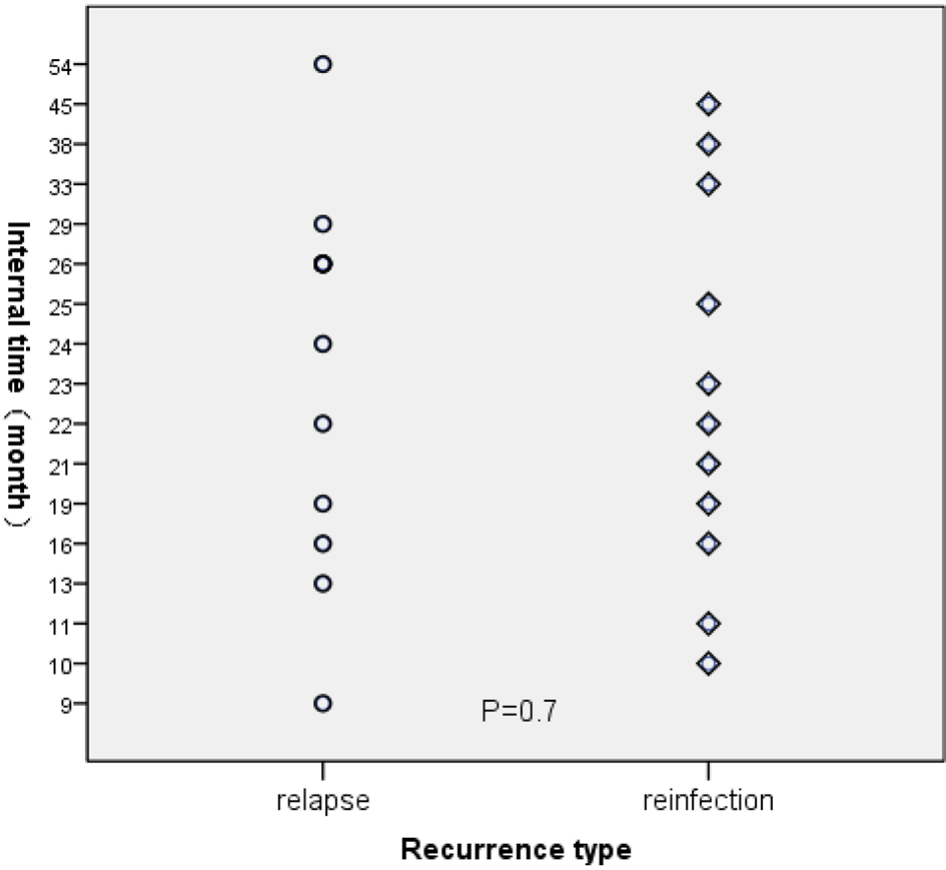
The interval time for recurrent TB.

For those 23 TB cases, the average interval time between the first and second episodes was 24.04 months (95%CI:19.37-28.71). Meanwhile, the average interval periods between the first and second episodes for the relapsed cases and re-infected cases were 24.17 and 23.91 months, respectively (*P*=0.7) (**Figure 3**).

We further compared drug susceptibility profiles between relapsed and re-infected TB cases. Out of those 12 relapsed cases, there were two pairs detected with inconsistent DST results, one exhibited change from isoniazid-resistance to MDR, while another rifampicin-resistant isolate was transformed to susceptible isolate in the second episode. Meanwhile, there were four inconsistent drug-susceptibility results among the re-infection group. In general, two susceptible isolates were replaced by MDR isolates, and one another susceptible isolate was replaced by isoniazid and streptomycin-resistant isolate. Besides, one MDR patient was found reverted to susceptible in the second episode (**Table 1**).

## Discussion

The emerging of recurrent TB posed a great challenge for the national TB elimination program in China. Previous studies indicated that recurrent TB patients required longer diagnostic delay time compared to new cases, which increased the opportunity of transmission (Nsofor et al., 2017; Xie et al., 2020). Meanwhile, the unfavorable treatment outcome and poor management were usually accompanied by the recurrence of TB (Nakanwagi-Mukwaya et al., 2013; Dedefo et al., 2019).

In this study, the rate of recurrence TB was up 2%, which was a little higher than that of Europe (Millet et al., 2013) and distinctly lower than that of India (Cardona et al., 2018). It seems that TB recurrence declined with the decreasing incidence of TB. However, it was still reported a reverse relationship between TB recurrence and incidence (Lambert et al., 2003).In Korea, the recurrence rate declined yearly from 2005-2010 along with an increased TB notification. In our study, we found that relapse and re-infection contributed equally to TB recurrence. A previous surveillance study conducted in London during 2002-2015 revealed that the rate of re-infection was much higher than that of relapse (Wingfield et al., 2019), but an opposite result was raised by another study carried out in Beijing, which indicated that recurrent TB was dominated by relapse cases (Nsofor et al., 2017). Several reasons could be responsible for such variation of the proportion of recurrence. First, the varied study observation length might result in different proportion of recurrence. Commonly, relapse occurred earlier than re-infection (Luzze et al., 2013), if the study tracked cases in a relatively shorter period, re-infection cases would be missed, resulting in a relatively lower re-infection rate (Marx et al., 2014) Besides, different genotyping methods, such as MIRU-VNTR and whole-genome sequencing (WGS), qualified as disparate discriminatory power that would make difference in classification of recurrence (Kruuner et al., 2002; Bryant et al., 2013b). Moreover, human immunodeficiency virus (HIV) and MDR, could increase the likelihood from infection to disease and generate more re-infection cases (Cox et al., 2006; Pettit et al., 2011). Therefore, the rate of reported relapse or re-infection varied widely in different studies. The relationship between the risk factors, such as age, gender, and cavitation on chest X-ray, and recurrence were controversial in past studies (Moosazadeh et al., 2015; Silva et al., 2017; Cudahy et al., 2020), in our study there was no significant association between those factors and different recurrence types.

In general, the rate of relapse due to inadequate treatment or ineffective human immunity is important to evaluate TB treatment regimen and patient management (Lambert et al., 2003). Considering the directly observed therapy (DOT) was fundamentally implemented in China for many years, the treatment outcome assessment might not be effective as expected. Currently, sputum smear was taken as one of the main methods to evaluate the treatment outcome of TB. However, most patients could not provide sputum samples at the end of treatment. Thus, treatment outcome was mainly assessed by chest X-ray examination. Meanwhile, previous studies had revealed that chest X-ray result was weakly related to bacteria grade before the treatment, and limited in the assessment of the outcome after treatment (Murthy et al., 2018; Lee et al., 2020). So a feasible and robust assessment of treatment efficacy should be established to solve such a dilemma.

Acquired drug resistance for relapsed TB should be concerned, especially for acquired MDR-TB. A previous study indicated recurrence of TB was a risk factor for rifampicin-resistance (Chen et al., 2019). In our study, there was an isoniazid- resistant TB (Hr-TB) developed into MDR-TB in the second episode of the relapse group. Nowadays, isoniazid-resistance detection was not prioritized for TB drug-resistance detection, where GeneXpert MTB/RIF was extensively used for rifampicin-resistant screening. Thus, most Hr-TB patients were not detected and prescribed a standard anti-TB regimen. A previous study had revealed that inappropriate treatment of Hr-TB would result in MDR as well (Murray et al., 2009). Meanwhile, the treatment failure and relapse rates for Hr-TB were significantly higher than drug-susceptible TB (Gegia et al., 2012; Gegia et al., 2017). Thus, we suggested drug susceptibility test of isoniazid should be carried out, and appropriate treatment regimens should be adopted for Hr-TB.

At the same time, a rifampicin-resistant TB prescribed with a 6-month treatment regimen reached a treatment success in the first episode of TB. The reason for prescribing the first-line anti-TB drugs because the culture-based anti-TB drug susceptibility test usually came out the results three months later. However, the clinical symptom and X-ray examination demonstrated an effective treatment under this 6-month treatment regimen. Thus, 6-month treatment was prescribed until a successful treatment was reached. Previous studies indicated first-line anti-TB drugs might be helpful for MDR-TB treatment in this region (Zheng et al., 2020)The second TB episode of this case was happened 54 months after successful treatment of the first episode as shown in **Table 1**, and the MIRU data demonstrated an identical genotype but the drug resistant testing demonstrated rifampicin-susceptible. A previous study had revealed drug resistant changes between the two episodes for relapsed TB cases (Zong et al., 2018).

On the other hand, re-infection from an exogenous pathogen is associated with the recent spread of TB which required high-quality public health programs to restrict transmission. Notably, re-infected cases harbored more MDR than relapsed cases, although there was no significant difference. Therefore, further interventions for re-infection should focus on the management of MDR patients, and early detection of drug-susceptible character for the clinical patient.

Several limitations should be mentioned. First, compared to the whole genome sequencing, the discrimination power was relatively lower for 24 loci MIRU-VNTR, the genetic diversity of relapse and re-infection cases might be overlapped, but MIRU-VNTR was still a major molecular method for strain identification. Second, identical genotypes caused by re-infection can’t be overlooked, especially those identical genotypes belong to the main clusters of the area. Third, the number of recurrent TB in this study was relatively small, the drug-resistant profile was limited to explore the transmission model of drug resistance for recurrent TB.

## Conclusion

This study illustrated that endogenous relapse and exogenous re-infection contributed equally to the recurrence of TB, while re-infection cases were more likely to exhibit MDR in a second episode. Distinguishing between relapse and re-infection would be very necessary for design a more efficient TB control strategy. Meanwhile, the acquired drug resistance for relapsed cases should be concerned as well, especially for the first incident of isoniazid resistance.

## Data Availability Statement

The raw data supporting the conclusions of this article will be made available by the authors, without undue reservation.

## Ethics Statement

The studies involving human participants were reviewed and approved by the institutional review board of Jiangsu province center for disease control and prevention. The patients/participants provided their written informed consent to participate in this study.

## Author Contributions

YS wrote the draft of the manuscript. CC designed and edited the manuscript. YS, HS, GL, YL, and YSL conducted the experiments. WL, LZ, and CC reviewed the data collection. All authors contributed to the article and approved the submitted version.

## Funding

This work was supported by the National Major Science & Technology Projects for Infectious Disease Control and Prevention [grant number 2018ZX10715002], Jiangsu Commission of Health [grant number M2020040], Center for Disease Control and Prevention of Jiangsu Province [grant number JKRC2016006]. The funders had no role in study design, data collection, and analysis, decision to publish, or preparation of the manuscript.

## Conflict of Interest

The authors declare that the research was conducted in the absence of any commercial or financial relationships that could be construed as a potential conflict of interest.
